# The rs1403543 Polymorphism of *AGTR2*, Which Encodes the Type-2 Angiotensin II Receptor, and Left Ventricular Mass in Polish Full-Term Newborns

**DOI:** 10.3390/genes16050518

**Published:** 2025-04-29

**Authors:** Iwona Gorący, Karol Miler, Klaudyna Lewandowska, Monika Rychel, Beata Łoniewska, Andrzej Ciechanowicz

**Affiliations:** 1 Department of Clinical and Molecular Biochemistry, Pomeranian Medical University, 70-111 Szczecin, Poland; iwona.goracy@pum.edu.pl (I.G.); kmiler01@gmail.com (K.M.); klaudyna.lewandowska@pum.edu.pl (K.L.); monika.rychel@gmail.com (M.R.); 2 Department of Neonatal Diseases, Pomeranian Medical University, 70-111 Szczecin, Poland; beata.loniewska@pum.edu.pl

**Keywords:** newborns, left ventricular mass, genetic polymorphism, type 2 receptor for angiotensin II

## Abstract

Background/Objectives: Left ventricular hypertrophy is a significant independent risk factor for increased cardiovascular morbidity and mortality. There are some reports indicating an association of rs1403543 (1675G>A) polymorphism in the *AGTR2* gene, which encodes the type-2 angiotensin II receptor, with left ventricular hypertrophy or increased left ventricular mass (LVM) in adults. The aim of this study was to analyze the possible association of the *AGTR2*:rs1403543 polymorphism with LVM in full-term Polish healthy newborns. Methods: The study group comprised 207 consecutive, full-term, healthy newborns. LVM was assessed, on the 3rd day after birth, from the M-mode echocardiographic measurements of left ventricular dimensions using the Penn convention, with the Huwez et al.-modified equation mode. The *AGTR2* polymorphism was identified by PCR-RFLP in genomic DNA extracted from cord blood leukocytes. Results: There were no significant differences in clinical and echocardiographic characteristics of male newborns in regard to the *AGTR2*:rs1403543 polymorphism. However, the LVM/body mass ratio in female newborns carrying at least one A allele (i.e., with genotype GA or AA) was significantly lower as compared to its value in reference (GG) homozygotes. In addition, in female newborns, the frequency of *AGTR2* genotypes with at least one A allele was significantly higher in the lower tertile of LVM/body mass or LVM/body surface area (calculated using the Mosteller formula) ratios as compared with upper tertiles. Conclusions: Our results suggest that the *AGTR2*:rs1403543 polymorphism may be associated with the physiological variability of cardiac mass in female newborns.

## 1. Introduction

Left ventricular hypertrophy (LVH) is recognized when the left ventricular mass index (LVMI), calculated as the left ventricular mass (LVM) divided *per* body surface area (BSA), is >95 g/m^2^ in women or >115 g/m^2^ in men [1,2]. A number of studies have demonstrated that LVH is a significant independent risk factor for increased cardiovascular morbidity and mortality and, therefore, a major public health burden, especially in light of an aging population [3]. The results of several studies have suggested that approximately 30% to 70% of cardiac mass variability can be explained by heredity [4,5,6,7,8].

The renin–angiotensin system (RAS) is both a major regulator of maternal–fetal health [9] as well as the crucial endocrine element in the regulation of renal and cardiovascular homeostasis [10]. One crucial element of this homeostasis is the action of angiotensin II (ANGII) as a ligand via two types of receptors. Stimulation of the type-1 angiotensin II receptor (AT1R) results, i.a., in an increase of blood pressure and in induction of cardiac hypertrophy and fibrosis, while activation of the type-2 receptor (AT2R) gives opposing effects [10].

The expression of AT2R is high in the neonatal heart and declines after birth (for review, see [11]). It is worth noting that the heart masses of 7-day-old and 14-day-old AT2R-knockout mice were significantly higher than those of age-matched control mice. However, the heart-to-body-mass ratio in AT2R-deficient mice in this early postnatal period was significantly lower than those of age-matched control mice [12]. In addition, the AT2R deficiency resulted in a stronger increase in blood pressure in response to infusion of angiotensin II [13,14,15]. This suggests that the stimulation of AT2R by angiotensin II affects postnatal cardiac growth, possibly via reducing body mass gain and lowering blood pressure [10,12].

The type-2 angiotensin II receptor in humans is encoded by the *AGTR2* gene located at chromosome Xq23. The common *AGTR2*:rs1403543 polymorphism gives a transition of guanine (G) to adenine (A) 1332 base pairs (bp) upstream of the translation start codon (-1332G>A), also known as 1675G>A (e.g., G-to-A transition located 1675 bp downstream of the transcription start) [16]. The dbSNP variant details give *AGTR2*:rs1403543 as being located at NC_000023.11:g.116170939G>A according to genome assembly GRCh38.p14 chr X and as having two transcripts with locations NM_000686.5:c.-95-29G>A and NM_001385624.1:c.-36+123G>A. In this article, we will refer to this single nucleotide variant as rs1403543 or 1675G>A. The A allele has a global ALFA allele frequency of 52%, and the reference G allele has 48% (a T allele being extremely rare; dbSNP) [17].

So far, only a few reports have focused on the analysis of the association between *AGTR2*:rs1403543 polymorphism and LVMI and/or LVH and, in addition, these reports have yielded contradictory results [18,19,20,21,22,23,24,25]. In 2001, Schmieder et al. showed, in a group of 120 young males of European descent, that hypertensive (but not normotensive) subjects hemizygous for the *AGTR2*:1675A allele had a greater LVMI as compared with hypertensives with the reference *AGTR2* variant (with the 1675G allele) [18]. One year later, in the GLAOLD (Glasgow Heart Scan Old) study of subjects aged 55–74 years, Herrmann et al. found that the frequency of *AGTR2*:1675A allele carriers was significantly higher in males with LVH than in those without left ventricular hypertrophy, and this effect was not observed in females [19]. In contrast, Alfakih et al. reported a significantly higher prevalence of GG homozygous females and G hemizygous males among a group with magnetic resonance imaging (MRI)-assessed LVH as compared to a group without LVH (heterozygous females were excluded from the analysis) [20]. Also, in EPOGH men (but not in women), LVMI increased significantly with higher sodium excretion (4.2 g/m^2^ per 100 mmol) in carriers of the reference (1675G) allele, with the opposite tendency in males with the *AGTR2*:1675A allele [21]. In 2007, Ott et al. reported a lower LVM in males with the 1675G variant who were particularly susceptible to LVM modification by increased salt intake [22]. In contrast, Orlowska-Baranowska et al. found no significant association of *AGTR2*:1675G>A polymorphism with LVH in Polish patients with aortic stenosis [23]. Huber et al. also revealed no significant association of this *AGTR2* variant with LVMI in treated patients with arterial hypertension in Germany [24]. However, in patients with hypertrophic cardiomyopathy, Carstens et al. reported an allele-dependent modulating effect of the *AGTR2*:rs1403543 polymorphism on LVH (with a decrease in wall thickness by ~0.5 mm with each 1675A allele) which was independent of such covariates as causal mutation of hypertrophic cardiomyopathy or blood pressure [25]. It has not escaped our notice that all the above-mentioned studies concerning the association of the *AGTR2*:rs1403543 polymorphism and LVM have been carried out in adults. However, it is worthy to underline that reliable evidence suggests that cardiovascular disease (CVD) begins early in life [26,27]. There have been reports of association of CVD risk factors in childhood with both increased cardiac growth in children [28,29] and LVH in adults [30,31]. Therefore, the aim of our study was to analyze the possible association of the *AGTR2*:rs1403543 polymorphism with echocardiographically measured left ventricular mass in full-term Polish healthy newborns.

## 2. Materials and Methods

### 2.1. Newborns

The criteria for newborn eligibility in the present study as well as newborn cord blood (500 μL) collection for the isolation of genomic DNA have been described before [32,33]. In brief, the study group comprised 207 healthy Polish newborns (96 females and 111 males), born between 38 and 40 weeks (inclusive) of gestation to healthy women with uncomplicated pregnancies. The sex of the newborn, body mass (BM; kg) and body length (BL; m) were taken from standard hospital records. Body surface area (BSA; m^2^) was calculated using the Mosteller formula [34]:

BSA = 0.0167 × (100 × BL)^0.5^ × BM^0.5^ (1)

All the studied newborns, on the third day after delivery, underwent transthoracic echocardiography as described previously [6,35]. Measurement techniques followed American Society of Echocardiography conventions. Left ventricular masses were estimated from echocardiographic left ventricular dimensions using the Penn convention, with the equation modified by Huwez et al. [36]:

LVM [g] = 1.04 × [(IVST [mm] + LVPWT [mm] + LVID [mm])^3^ − LVID [mm]^3^] (2)

where IVST, LVPWT and LVID denote interventricular septal thickness, left ventricular posterior wall thickness and left ventricular internal dimension, respectively. To give standardized parameters, the left ventricular mass was divided by body mass (LVM/BM, g/kg), body length (LVM/BL, g/m) or body surface area (LVM/BSA, g/m^2^). The study was conducted in accordance with the latest Declaration of Helsinki (2024) and was approved by the bioethics committee at the Pomeranian Medical University in Szczecin, Poland. Parental informed consent for newborn control data was obtained for each newborn.

### 2.2. AGTR2:rs1403543 Genotyping

Genomic DNA was isolated from cord blood as described previously [32,33]. For the genotyping of the *AGTR2*:rs1403543 polymorphism, a PCR-RFLP (polymerase chain reaction–restriction fragment length polymorphism) method was applied with 5′-GGAAAGTAGAACATACATTAAATG-3′ as the forward primer, and with 5′-CCTGTAAGAGAA ACAGCAGCTAAAGAATT′-3′ as the reverse primer (both primers from TIB MOL BIOL, Poznań, Poland). The *AGTR2* amplicons were subsequently digested with the EcoRI restriction enzyme (Thermo Fisher Scientific, Waltham, MA, USA). The PCR product was cut into fragments of 95 base pairs (bp) and 25 bp if the 1675G reference allele was present or remained uncut (with an amplicon of 120 bp in length) with the 1675A allele. Both electrophoretic separation of the restriction products and the verification of PCR-RFLP results were carried out as described before [33].

### 2.3. Statistical Analyses

Normality of the quantitative data was assessed by Kolmogorov–Smirnov or Lilliefors tests. As most quantitative variables were not normally distributed, all are presented as medians with minimum and maximum values. Quantitative data were compared using Mann–Whitney tests or Kruskal–Wallis tests, if necessary. Categorical data were assessed using chi-squared tests. Statistical significance was defined as *p* < 0.05. All data were analyzed using a data analysis software system (Statistica, version 13, TIBCO software, Palo Alto, CA, USA).

## 3. Results

There were 28 (29.2%) GG homozygotes, 45 (46.9%) GA heterozygotes and 23 (23.9%) AA homozygotes among female newborns, and the frequency of the *AGTR2*:1675A allele in this group was 47.4%. The *AGTR2*:rs1403543 genotype distribution in female newborns was consistent with the Hardy–Weinberg equilibrium (*p* = 0.557). There were 58 (52.2%) male newborns hemizygous for the *AGTR2*:1675G reference allele and 53 (47.8%) male newborns with the 1675A allele.

Clinical and echocardiographic newborn characteristics are shown in Table 1. There were no significant differences between females and males in terms of body length, left ventricular dimensions (IVST, LVPWT and LVID) or LVM and LVM indices (LVM/BM, LVM/BL and LVM/BSA). Only body mass (BM) and body surface area (BSA) in male newborns were significantly higher than in females.

**Figure 1:**
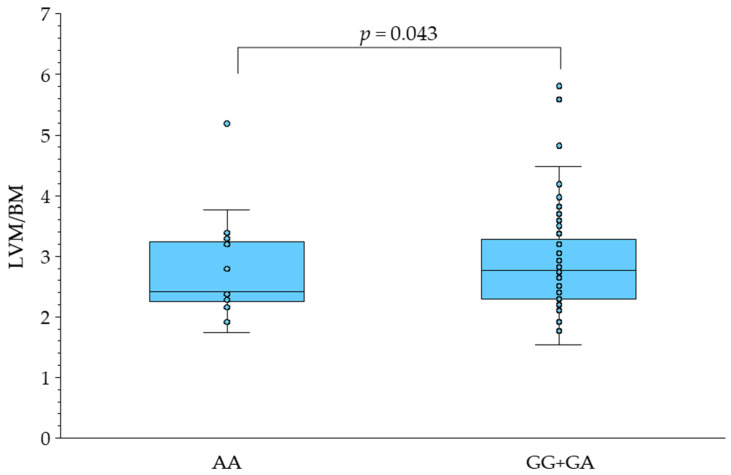
LVM/BM index in female newborns in regard to *AGTR2*:rs1403543 polymorphism. Box plots show medians (m), interquartile range (q0.25 to q0.75), range (whiskers) between q × 0.25 − 1.5 × (m – q × 0.25) to q × 0.75 + 1.5 × (q × 0.75 − m) and outliers.

There were no significant differences in the values of clinical and echocardiographic variables in female newborns in regard to *AGTR2*:rs1403543 genotype except for LVM/BM. The LVM/BM in female newborns carrying at least one 1675A allele (with a GA or AA genotype) was significantly lower as compared to its value in females homozygous for the reference *AGTR2* allele (Table 2 and Figure 1).

Other than for BSA, there were no significant differences in clinical and echocardiographic characteristics of male newborns in regard to *AGTR2*:rs1403543 hemizygosity. The BSA in male newborns hemizygous for the *AGTR2*:1675A allele was significantly lower as compared to males with the reference variant (with the 1675G allele) (Table 3). Further, separately for females and males, the distributions of *AGTR2*:rs1403543 polymorphism in tertiles of LVM indices were analyzed. No significant associations were found both in females or males between the distributions of *AGTR2*:1675G>A variants or tertiles of LVM indices except for LVM/BL and LVM/BSA in female newborns (Table 4 or Table 5, respectively). The frequency of *AGTR2* genotypes with at least one *AGTR2*:1675A allele was significantly higher for both LVM/BM and LVM/BSA in female newborns from the lower tertile as compared with subjects from the upper tertile (Table 4).

Finally, after exclusion of GA heterozygous female newborns (due to the presumed effect of random inactivation of the one of the X chromosomes in females), we assessed the differences between GG/G and AA/A subjects (GG homozygous females + G hemizygous males versus AA homozygous females + A hemizygous males).

After the exclusion of GA heterozygotes, no significant differences between female newborns and male newborns were found in the values of body length, left ventricular dimensions or LVM and LVM indices. Only the body mass (BM) and body surface area (BSA) in male newborns were significantly higher as compared to females (Table 6).

There were no significant differences in the values of clinical and echocardiographic variables between subjects carrying the 1675G allele and carriers of the 1675A variant (Table 7).

No significant differences in the frequency distributions of the *AGTR2* polymorphism with regard to the tertiles of LVM indices were found in the comparison of GG/G with AA/A newborns (Table 8).

## 4. Discussion

This study has focused on the analysis of the association between the *AGTR2*:rs1403543 polymorphism and left ventricular mass (LVM) in full-term healthy newborns. The results revealed no significant association of *AGTR2* variants with LVM indices in male newborns, but they were associated with a lower LVM in female newborns carrying the 1675A allele. Our results with female newborns are consistent with those of a previous study by Alfakih et al. [20], who reported both a lower LVM index in AA/A subjects (i.e., in AA females and A males) than in GG/GA females and an excess of GG/G subjects among a group of patients with MRI-determined left ventricular hypertrophy. In addition, in a family cohort of patients with hypertrophic cardiomyopathy (HCM), Carstens et al. found a decrease in average wall thickness of ~0.5 mm with each *AGTR2*:1675A allele independent of the effects of the primary HCM causal mutation, blood pressure and other hypertrophy covariates [24]. However, it is also worthy to note that other studies either did not confirm in women an association of the 1675A *AGTR2* allele with LVM [21] or LVH [19,23,25] or revealed such association in males only [18,19,22]. In contrast, Kuznetsova et al. found that LVMI increase (related to higher salt intake) was significantly higher in males with the 1675G variant as compared to males with the A allele [21]. Therefore, taking into consideration the above discrepancies, we decided to carry out our study in healthy newborns born at term. The reasons for this are that newborns have a relative lack of exposure (or at least no long-term exposure) to confounding environmental factors, coexisting diseases or medication, and full-term healthy newborns are a more appropriate target group to study the association between genetic polymorphisms and cardiovascular intermediate phenotypes such as cardiac mass [37]. Last, but not least, an argument in favor of performing an association analysis of X-chromosome-located genes (such as *AGTR2*) in female newborns is the low frequency of nonrandom (skewed) X-chromosome inactivation (XCI) in female newborns [38] (although, even so, in one analysis, heterozygotes were excluded). In addition, for the rs1403543 biallelic polymorphism of the *AGTR2* gene located at chromosome X, women have three possible genotypes (GG, GA or AA), while men are hemizygous either for 1675G or the 1675A allele. Therefore, to ensure equal gene dosage, we carried out an association analysis of *AGTR2* polymorphism with LVM indices in the combined group (consisting of female and male newborns) after exclusion of heterozygous female newborns as in the study by Alfakih et al. [20].

The *AGTR2* gene spans approximately 5 kb and consists of three exons. Only the third exon encompasses the complete protein-coding sequence of 363 amino acids of the G-protein-coupled receptor [39]. The *AGTR2* gene 5′-flanking region contains typical eukaryotic promoter sequence motifs but also an Interferon Consensus Sequence Binding Protein (ICSBP) site and a putative Embryonal, Long Terminal Repeat Binding Protein (ELP) site what suggests a possible unusual regulation of *AGTR2* expression [39]. In addition, Warnecke et al. have indicated that sequence elements in intron 1 of the *AGTR2* gene are necessary for its efficient transcription [40]. Kuznetsova et al. have suggested that the *AGTR2*:rs1403543 polymorphism located in intron 1 is probably functional [21]. However, the molecular effects of *AGTR2*:1675G>A transition have been not fully elucidated and remain unclear. In 1999, Nishimura et al. pointed out that the *AGTR2*:rs1403543 transition is located within the lariat branchpoint motif of intron 1, which disrupts the efficiency of mRNA splicing. The authors also reported that cDNA from code containing the A allele consists of all three exons but cDNA from code with the G allele is 60 nucleotides shorter due to a lack of exon 2. In addition, the amount of mRNA transcribed from the G allele code was significantly lower as compared with mRNA from the A allele code [41]. However, six years later Warnecke et al. indicated that the *AGTR2*:1675G>A polymorphism modulates receptor protein expression rather than mRNA splicing. Using in vitro transfection assays with a luciferase reporter gene, the authors revealed that subjects carrying the 1675G allele may express higher levels of the type-2 angiotensin II receptor protein than those with the A allele [42]. It is not possible to rule out that the intronic rs1403543 polymorphism affects transcription of this gene per se but, additionally, it is in close linkage disequilibrium with other *AGTR2* variants of real functional importance, as discussed below.

Our analyses (using https://www.ensembl.org/Homo_sapiens/Tools/LD, accessed on 3 April 2025) in European-descent populations from the 1000 Genomes (1KG) Project have indicated that rs14003543 is in tight (r^2^ 0.785, D′ 0.957), very tight (r^2^ 0.842, D′ 0.999) or even in complete (r^2^ 1.000, D′ 1.000) linkage disequilibrium (LD) with the *AGTR2*:rs11091046 polymorphism (GRCh38.p14 chr X:NC_000023.11:g.116173873A>C (dbSNP); also known as 3123C>A or 4599C>A [37] for TSI (Toscani in Italy), CEU (Utah Residents with Northern and Western European ancestry) or GBR (British in England and Scotland), respectively). The rs11091046 polymorphism is located in the 3′-untranslated region of *AGTR2* exon 3, at a microRNA-response-element position possibly corresponding to the hsa-miR-208a-5p and hsa-miR-208b-5p binding sites [43]. The miR-208a and miR-208b elements are encoded in introns of the myosin heavy chain 6 gene (*MYH6*) or the myosin heavy chain 7 gene (*MYH7*), respectively [44,45]. Van Rooij et al. showed that miR-208a is required, i.a., for cardiomyocyte hypertrophy and fibrosis [44], while miR-208b is rather involved in the control of muscle myosin content [45]. Therefore, Yvert et al. suggested that *AGTR2* expression may be influenced partly by miR-208 a- and/or b-5p and that the C allele of the *AGTR2*:rs11091046 polymorphism (in linkage disequilibrium with the 1675A allele) can reduce the binding affinity of miRNA to mRNA, leading to an increased amount of type-2 angiotensin II receptor protein [43]. It is also worthy to note that the *AGTR2*:rs11091046 polymorphism has been previously found to be associated with several cardiovascular phenotypes such as acute myocardial infarction [46], hypertension [47] or hypertrophic cardiomyopathy [48]. In the latter study, carried out with 103 genetically independent subjects with HCM, Deinum et al. showed a significant decrease in LVMI with the number of AGTR2:3123C alleles in women only but no association of this index with rs11091046 in men [48].

A major limitation of our study is its relatively low statistical power mainly due to a relatively small sample size (96 female newborns and 111 male newborns). Such limited numbers of subjects in our study were due to the fact that the recruitment of newborns to the study had been completed several years ago, and good-quality genomic DNA samples remained only in these 207 newborns for which we also had the results of ultrasound measurements of heart dimensions. To further depict the issue, we have computed (using the Open Epi tool, version 3.01, a free and open-source software available at www.openepi.com, accessed on 12 February 2025) the minimum sample size of male newborns for 80% statistical power and 5% type I error rate with the assumptions that the ratio of G to A hemizygotes is equal to 1.09 (58/53) and LVM/BL is lower than 15.5 g/m in 29.3% (17 of 58) of G subjects and in 37.7% (20 of 53) of A subjects (Table 5). Under the above assumptions, the minimum sample size necessary varied from 992 to 1039 male newborns (517–542 G subjects and 475–497 A subjects). On the other hand, the sample size in our study (*n* = 211) was even greater than in four of eight previously published reports focusing on association analyses of *AGTR2*:rs14003543 or *AGTR2*:rs11091046 with LVM or LVH [18,19,20,21,22,23,24,25]. It is also worth noting that the number of female newborns in our study (*n* = 96) was greater than in the study by Alfakih et al. (*n* = 72) or by Deinum et al. (*n* = 40), who previously revealed the association of lower LVM in women with the 1675A allele [20] or with the 3123C variant [48], respectively.

## 5. Conclusions

The results of our study suggest that the *AGTR2*:rs1403543 polymorphism may be associated with physiological variability in cardiac mass in female newborns. However, we are fully aware that the inclusion of *AGTR2* polymorphism analysis into a cardiovascular risk assessment will require prior prospective longitudinal studies in much larger groups of newborns of specific ethnic origin, taking into account both the period and the degree of exposure to cardiovascular risk factors.

## Figures and Tables

**Table 1:** Clinical and echocardiographic characteristics of the newborns with regard to sex.

Variable	All Newborns (*n* = 207)	Female Newborns (*n* = 96)	Male Newborns (*n* = 111)	*p*
	Median (Minimum–Maximum)			
BM [kg]	3.43 (1.87–5.09)	3.30 (1.87–4.48)	3.51 (2.36–5.09)	0.003
BL [m]	0.56 (0.44–0.63)	0.55 (0.46–0.63)	0.56 (0.44–0.63)	0.055
BSA [m^2^]	0.230 (0.158–0.289)	0.227 (0.158–0.270)	0.232 (0.170–0.289)	0.006
IVST [mm]	3.7 (2.4–6.3)	3.6 (2.4–6.0)	3.7 (2.6–6.3)	0.592
LVPWT [mm]	2.8 (1.1–5.8)	2.5 (1.3–5.0)	2.8 (1.1–5.8)	0.309
LVID [mm]	18.3 (15.0–25.0)	18.1 (15.0–25.0)	18.4 (15.6–23.5)	0.168
LVM [g]	9.2 (4.5–21.9)	8.8 (4.5–19.3)	9.4 (4.5–21.9)	0.062
LVM/BM [g/kg]	2.8 (1.5–6.4)	2.7 (1.5–5.8)	2.8 (1.6–6.4)	0.804
LVM/BL [g/m]	16.5 (8.5–35.9)	16.0 (9.0–33.3)	17.1 (8.5–35.9)	0.108
LVM/BSA [g/m^2^]	40.5 (22.2–90.6)	39.4 (22.2–80.7)	41.7 (22.4–90.6)	0.384

Legend: BM = body mass; BL = body length; BSA = body surface area; IVST = interventricular septal thickness; LVPWT = left ventricular posterior wall thickness; LVID = left ventricular internal dimension; LVM = left ventricular mass. The *p* values are from Mann–Whitney tests. Parameter values given to 1 decimal place (d.p.), apart from BM/BL (2 d.p.) and BSA and *p*-values to 3 d.p.

**Table 2:** Clinical and echocardiographic characteristics of the female newborns in regard to the *AGTR2*:rs1403543 (1675G>A) polymorphism.

Variable	GG (*n* = 28)	GA (*n* = 45)	AA (*n* = 23)	*p* _K-W_	*p* _D_	*p* _R_
	Median (Minimum–Maximum)					
BM [kg]	3.26 (1.87–4.30)	3.30 (2.30–4.22)	3.50 (2.70–4.48)	0.338	0.377	0.158
BL [m]	0.55 (0.46–0.63)	0.55 (0.47–0.62)	0.55 (0.50–0.60)	0.761	0.276	0.463
BSA [m^2^]	0.225 (0.158–0.270)	0.225 (0.173–0.270)	0.230 (0.194–0.268)	0.393	0.491	0.179
IVST [mm]	3.8 (2.8–6.0)	3.4 (2.4–4.8)	3.7 (2.4–6.0)	0.085	0.069	0.603
LVPWT [mm]	2.8 (1.7–5.0)	2.5 (1.3–3.8)	2.5 (1.5–5.0)	0.202	0.075	0.602
LVID [mm]	18.0 (15.0–21.4)	18.5 (15.0–21.2)	17.6 (16.0–25.0)	0.298	0.939	0.156
LVM [g]	8.9 (5.6–17.6)	8.8 (4.5–15.3)	8.2 (5.4–19.3)	0.331	0.138	0.619
LVM/BM [g/kg]	2.9 (1.8–5.8)	2.7 (1.5–4.2)	2.4 (1.7–5.2)	0.112	0.043	0.233
LVM/BL [g/m]	16.6 (11.0–32.0)	16.1 (9.0–26.4)	15.1 (10.0–33.3)	0.229	0.087	0.577
LVM/BSA [g/m^2^]	40.8 (26.6–80.7)	39.8 (22.2–63.1)	35.7 (26.2–78.8)	0.193	0.076	0.361

Legend: For abbreviations and numbers of decimal places see Table 1. The *p* _K-W_ values are from Kruskal–Wallis tests, and the *p* _D_ or *p* _R_ values are from Mann–Whitney tests in dominant or recessive mode of inheritance for the *AGTR2*:1675A allele (AA + GA versus GG, or AA versus GG + GA, respectively).

**Table 3:** Clinical and echocardiographic characteristics of the male newborns in regard to the *AGTR2*:rs1403543 (1675G>A) polymorphism.

Variable	G (*n* = 58)	A (*n* = 53)	*p*
	Median (Minimum–Maximum)		
BM [kg]	3.64 (2.36–5.09)	3.45 (2.73–4.40)	0.073
BL [m]	0.57 (0.44–0.63)	0.56 (0.47–0.61)	0.071
BSA [m^2^]	0.242 (0.170–0.289)	0.229 (0.196–0.269)	0.047
IVST [mm]	3.8 (2.0–6.3)	3.7 (2.7–5.6)	0.947
LVPWT [mm]	2.8 (2.0–4.3)	2.8 (1.1–5.8)	0.270
LVID [mm]	18.6 (15.6–23.5)	18.0 (15.6–23.2)	0.307
LVM [g]	9.8 (5.0–21.9)	9.4 (4.5–19.0)	0.392
LVM/BM [g/kg]	2.8 (1.8–6.4)	2.7 (1.6–4.7)	0.947
LVM/BL [g/m]	17.4 (9.2–36.9)	16.9 (8.5–33.4)	0.567
LVM/BSA [g/m^2^]	41.8 (25.0–90.6)	41.2 (22.4–72.8)	0.849

Legend: For abbreviations and number of decimal places, see Table 1.

**Table 4:** Frequency distributions of *AGTR2*:rs1403543 genotypes in female newborns in regard to tertiles of left ventricular mass (LVM) indices.

Variable	Tertile	GG *n* (%)	GA *n* (%)	AA *n* (%)	*p*	*p* _D_	*p* _R_
					_LT_ vs. _MT_ vs. _UT_ _(LT_ vs. _UT)_	_LT_ vs. _MT_ vs. _UT_ _(LT_ vs. _UT)_	_LT_ vs. _MT_ vs. _UT_ _(LT_ vs. _UT)_
	Lower (<2.4)	6 (19)	15 (47)	11 (34)	0.179 (0.150)	0.155 (0.055)	0.202 (0.266)
LVM/BM [g/kg]	Middle (2.4–3.0)	9 (28)	18 (56)	5 (16)			
	Upper (>3.0)	13 (41)	12 (38)	7 (22)			
	Lower (<14.7)	5 (16)	17 (53)	10 (31)	0.226 (0.138)	0.114 (0.047)	0.337 (0.578)
LVM/BL [g/m]	Middle (14.7–18.4)	11 (34)	16 (60)	5 (16)			
	Upper (>18.4)	12 (38)	12 (38)	8 (25)			
	Lower (<35.6)	5 (16)	17 (53)	10 (31)	0.315 (0.139)	0.114 (0.047)	0.475 (0.396)
LVM/BSA [g/m^2^]	Middle (35.6–44.6)	11 (34)	15 (47)	6 (19)			
	Upper (>44.6)	12 (38)	13 (41)	7 (22)			

Legend: For abbreviations see Table 1. The *p*, *p* _D_ or *p* _R_ values are from chi-squared tests for comparisons of GG versus GA versus AA and for comparisons in dominant or recessive mode of inheritance for the *AGTR2*:1675A allele (AA + GA versus GG, or AA versus GA + GG, respectively). LT versus MT versus UT or LT versus UT are for comparisons of lower tertile versus middle tertile versus upper tertile or for comparisons of lower tertile versus upper tertile, respectively. Parameter values given to 0 decimal places.

**Table 5:** Frequency distributions of *AGTR2*:rs1403543 variants in hemizygous male newborns in regard to tertiles of left ventricular mass indices.

Variable	Tertile	G *n* (%)	A *n* (%)	*p* _LT_ vs. _MT_ vs. _UT_	*p* _LT_ vs. _UT_
	Lower (<2.5)	22 (60)	15 (40)		
LVM/BM [g/kg]	Middle (2.5–3.0)	15 (40)	22 (60)	0.212	0.814
	Upper (>3.0)	21 (57)	16 (43)		
	Lower (<15.5)	17 (46)	20 (54)		
LVM/BL [g/m]	Middle (15.5–18.9)	19 (51)	18 (49)	0.504	0.244
	Upper (>18.9)	22 (60)	15 (40)		
	Lower (<37.4)	20 (54)	17 (46)		
LVM/BSA [g/m^2^]	Middle (37.4–45.4)	16 (43)	21 (57)	0.364	0.639
	Upper (>45.4)	22 (60)	15 (40)		

Legend: For abbreviations, see Table 1. The *p* values are from chi-squared tests for comparisons of lower tertile versus middle tertile versus upper tertile or lower tertile versus upper tertile (LT versus MT versus UT or LT versus UT, respectively). Parameter values given to 0 decimal places; *p*-values to 3 decimal places.

**Table 6:** Clinical and echocardiographic characteristics of the newborns with regard to sex after exclusion of *AGTR2* GA heterozygous females.

Variable	Female Newborns (*n* = 28 + 23)	Male Newborns (*n* = 111)	*p*
	Median (Minimum–Maximum)		
BM [kg]	3.32 (1.87–4.48)	3.51 (2.36–5.09)	0.028
BL [m]	0.55 (0.46–0.63)	0.56 (0.44–0.63)	0.195
BSA [m^2^]	0.230 (0.158–0.270)	0.230 (0.170–0.290)	0.049
IVST [mm]	3.7 (2.4–6.0)	3.7 (2.6–6.3)	0.430
LVPWT [mm]	2.5 (1.5–5)	2.8 (1.1–5.8)	0.894
LVID [mm]	17.8 (15.0–25.0)	18.4 (25.6–23.5)	0.065
LVM [g]	8.7 (5.4–19.3)	9.5 (4.5–21.9)	0.360
LVM/BM [g/kg]	2.7 (1.7–5.8)	2.8 (1.6–6.4)	0.762
LVM/BL [g/m]	15.9 (10.0–33.3)	17.1 (8.5–35.9)	0.489
LVM/BSA [g/m^2^]	38.9 (26.2–80.8)	41.7 (22.4–90.6)	0.857

Legend: For abbreviations and number of decimal places, see Table 1.

**Table 7:** Clinical and echocardiographic characteristics of the newborns in regard to *AGTR2*:rs1403543 (1675G>A) after exclusion of GA heterozygous females.

Variable	GG + G (*n* = 28 + 58)	AA + A (*n* = 23 + 53)	*p*
	Median (Minimum–Maximum)		
BM [kg]	3.46 (1.87–5.09)	3.47 (2.70–4.48)	0.563
BL [m]	0.56 (0.44–0.63)	0.56 (0.47–0.61)	0.282
BSA [m^2^]	0.230 (0.158–0.290)	0.230 (0.194–0.270)	0.357
IVST [mm]	3.8 (2.6–6.3)	3.7 (2.4–6.0)	0.704
LVPWT [mm]	2.8 (1.7–5.0)	2.7 (1.1–5.8)	0.102
LVID [mm]	18.4 (15–23.5)	18.0 (15.6–25.0)	0.206
LVM [g]	9.5 (5.0–21.9)	9.2 (4.5–19.3)	0.207
LVM/BM [g/kg]	2.8 (1.8–6.4)	2.7 (1.6–5.2)	0.357
LVM/BL [g/m]	17.2 (9.2–35.9)	16.2 (8.5–33.4)	0.278
LVM/BSA [g/m^2^]	41.7 (25.0–90.6)	40.1 (22.4–78.8)	0.310

Legend: For abbreviations and number of decimal places, see Table 1.

**Table 8:** Frequency distributions of *AGTR2*:rs1403543 variants in GG homozygous females and G hemizygous males and in AA homozygous females and A hemizygous males in regard to tertiles of total kidney volumes.

Variable	Tertile	GG + G *n* (%)	AA + A *n* (%)	*p* _LT_ vs. _MT_ vs. _UT_	*p* _LT_ vs. _UT_
	Lower (<2.4)	27 (50)	27 (50)		
LVM/BM [g/kg]	Middle (2.4–3.0)	27 (50)	27 (50)	0.538	0.334
	Upper (>3.0)	32 (59)	22 (41)		
	Lower (<15.4)	25 (46)	29 (54)		
LVM/BL [g/m]	Middle (15.4–19.0)	29 (54)	25 (46)	0.400	0.177
	Upper (>19.0)	32 (59)	22 (41)		
	Lower (<37.0)	25 (46)	29 (54)		
LVM/BSA [g/m^2^]	Middle (37.0–45.5)	28 (52)	26 (48)	0.297	0.123
	Upper (>45.5)	33 (61)	21 (39)		

Legend: For abbreviations, see Table 1. The *p* values are from chi-squared tests for comparisons of lower tertile versus middle tertile versus upper tertile or lower tertile versus upper tertile (LT versus MT versus UT or LT versus UT, respectively). For numbers of decimal places, see Table 5.

## Data Availability

The data are available from the corresponding author upon reasonable request.

## Abbreviations

The following abbreviations are used in this manuscript:

*AGTR2*	Type-2 Angiotensin II Receptor gene
LVM	Left Ventricular Mass
PCR	Polymerase Chain Reaction
RFLP	Restriction Fragment Length Polymorphism
BM	Body Mass
BL	Body Length
BSA	Body Surface Area
LVH	Left Ventricular Hypertrophy
RAS	Renin–Angiotensin System
ANGII	Angiotensin II
AT1R	Type-1 Angiotensin II Receptor
AT2R	Type-2 Angiotensin II Receptor
LVMI	Left Ventricular Mass Index
GLAOLD	Glasgow Heart Scan Old
MRI	Magnetic Resonance Imaging
EPOGH	European Project On Genes in Hypertension
CVD	Cardiovascular Disease
IVST	Interventricular Septal Thickness
LVPWT	Left Ventricular Posterior Wall Thickness
LVID	Left Ventricular Internal Dimension
ICSBP	Interferon Consensus Sequence Binding Protein
ELP	Embryonal, Long Terminal Repeat Binding Protein
cDNA	Complementary deoxyribonucleic acid
mRNA	Messenger ribonucleic acid
D′	The difference between the observed and the expected frequency of a given haplotype
r^2^	The correlation between a pair of loci
LD	Linkage Disequilibrium
dbSNP	Single-Nucleotide Polymorphism database
TSI	Toscani in Italy
CEU	Utah Residents with Northern and Western European ancestry
GBR	British in England and Scotland
*MYH6*	Myosin Heavy Chain 6 gene
*MYH7*	Myosin Heavy Chain 7 gene
Hsa-miR	*Homo sapiens* microRNA (messenger ribonucleic acid)
